# Interaction of ciliary disease protein retinitis pigmentosa GTPase regulator with nephronophthisis-associated proteins in mammalian retinas

**Published:** 2010-07-17

**Authors:** Carlos A. Murga-Zamalloa, Nimit J. Desai, Friedhelm Hildebrandt, Hemant Khanna

**Affiliations:** 1 Department of Ophthalmology and Visual Sciences and Howard Hughes Medical Institute, University of Michigan, Ann Arbor, MI; 2 Department of Pediatrics and Human Genetics, University of Michigan, Ann Arbor, MI

## Abstract

**Purpose:**

Retinitis pigmentosa GTPase regulator (RPGR) is a cilia-centrosomal protein that frequently mutates in X-linked retinal degeneration and associated disorders. RPGR interacts with multiple ciliary proteins in the retina. Perturbations in the assembly of RPGR complexes are associated with retinal degeneration. This study was undertaken to delineate the composition and dissection of RPGR complexes in mammalian retinas.

**Methods:**

Immunoprecipitation of RPGR from ciliary fraction of bovine retina was performed, followed by mass spectrometry analysis. The glutathione S-transferase pull-down assay was performed to validate the interaction. Immunodepletion experiments were performed to dissect the partitioning of RPGR in different protein complexes in mammalian retinas.

**Results:**

We found that RPGR associates with a ciliary protein nephrocystin-4 (nephroretinin; NPHP4) that is mutated in nephronophthisis (NPH) and RP (Senior-Løken syndrome). This association is abolished in the *Rpgr*-knockout mouse retina. The RCC1-like domain of RPGR interacts with the N-terminal 316 amino acids of NPHP4. In the retina, RPGR also associates with NPHP1, an NPHP4-interacting protein; RPGR interacts directly with amino acids 243–586 of NPHP1. We further show that, in the retina, RPGR associates with and is partitioned in at least two different complexes with NPHP-associated proteins, (i) NPHP1, NPHP2, and NPHP5, and (ii) NPHP4, NPHP6, and NPHP8.

**Conclusions:**

RPGR may regulate some complexes with NPHP proteins in the mammalian retina. The disruption of these complexes may contribute to the pathogenesis of retinal degeneration in X-linked RP and associated ciliary diseases.

## Introduction

Primary or sensory cilia are microtubule-based extensions of the plasma membrane that are found in almost all cell types [1,2]. They regulate diverse cellular processes, including signal transduction, sensory perception, and protein trafficking [3]. Assembly of cilia occurs by a conserved process called intraflagellar transport (IFT) [4]. During IFT, precursor moieties are assembled at the base of the cilia (basal bodies) and transported in anterograde and retrograde directions by the kinesin-II and dynein motor subunits. The transport is facilitated by the multiple IFT polypeptides. Defects in cilia assembly or function are associated with multi-systemic disorders, including Senior-Løken syndrome (SLSN), Bardet-Biedl syndrome, Joubert syndrome (JBTS), and Meckel-Gruber syndrome [5,6].

Retinitis pigmentosa (RP; OMIM 268000), a degenerative disease of the photoreceptors, is frequently associated with ciliary dysfunction. RP is a clinically and genetically heterogeneous group of disorders characterized by severe vision loss and blindness [7]. X-linked RP (XLRP) is a common form of RP, accounting for 10%–15% of all RP cases [8,9]. Clinical manifestations of XLRP usually include night blindness due to rod photoreceptor dysfunction, followed by loss of cone function and blindness by the fifth decade of life [8,9]. Some female carriers also exhibit severe retinal defects.

RP3, a major locus for XLRP, encodes for the retinitis pigmentosa GTPase regulator (*RPGR*) gene [10,11]. Mutations in *RPGR* account for 70%–80% of XLRP and ~20% of simplex RP cases [12,13]. Some *RPGR* patients exhibit extra-retinal phenotypes, including hearing dysfunction, sperm defects, respiratory infections, and primary cilia dyskinesia [14–16]. The *RPGR* gene undergoes extensive alternative splicing and expresses multiple protein isoforms in the retina [17–20]. Most RPGR isoforms contain a common N-terminal domain encoded by exons 1–15, which encompass an RCC1-like domain (RLD; encoded by exons 2–11). The originally described constitutive isoforms of RPGR are encoded by exons 1–19 and account for ~20% of XLRP patients with no known mutations in exons 16–19. Later studies revealed another isoform of RPGR that contains an alternative terminal exon ORF15 (encompasses part of intron 15). Mutations in exon ORF15 account for additional 50%–60% of XLRP patients.

The RLD of RPGR is thought to be the functional domain based on its homology to RCC1 and its involvement in interaction with other proteins. RPGR and RPGR-RLD predominantly localize to primary cilia and photoreceptor connecting cilium (CC) [19,21], which is a conduit for trafficking of proteins from the inner segment to the photosensitive outer segment [22]. An *Rpgr*-knockout (*Rpgr*-ko; deletion of exons 4–6 in RLD) mouse mutant [23], which was later shown to carry a hypomorphic allele of *Rpgr* [19], exhibits delayed onset photoreceptor degeneration and mistrafficking of cone opsins. In addition, two canine models of RPGR mutation have been reported [24]. These animal models exhibit disparate phenotypes depending upon the type of mutation. Despite extensive efforts, it is still not clear how RPGR regulates photoreceptor function or how mutations in RPGR cause retinal degenerative disease.

Identification of RPGR-interacting proteins has played a key role in understanding its function. RPGR interacts with several ciliary and transport proteins in the retina, including intraflagellar transport protein IFT88/Polaris and RPGR-interacting protein 1 (RPGRIP1) [19,25]. In addition, RPGR associates with NPHP proteins mutated in renal retinal syndromes, including SLSN and JBTS [26–29]. For example, RPGR exists in complex with NPHP5 (or IQ domain containing calmodulin binding protein [IQCB1]; SLSN), centrosomal protein of 290 kDa (CEP290)/NPHP6 (Leber congenital amaurosis, SLSN, JBTS), and NPHP8/RPGRIP1-like (RPGRIP1L; mutated in JBTS and Meckel-Gruber syndrome) in the retina [30–32]. Notably, hypomorphic mutations in NPHP6 and NPHP8, which are associated with relatively early-onset photoreceptor degeneration [26–29,33–35], disrupt their association with RPGR [30,31]. Based on these observations, we hypothesize that RPGR-containing multiprotein complexes play a key role in facilitating photoreceptor protein trafficking.

To elucidate the precise role of RPGR in regulating ciliary transport, it is important to identify and characterize the components of the RPGR-interaction network in the retina. Using co-immunoprecipitation (IP) and mass spectrometry analysis, here we report that RPGR binds to NPHP1 and NPHP4. Using serial immunodepletion, we also found that the RPGR-NPHP interaction network can be divided into at least two distinct complexes: the first complex constitutes NPHP1, NPHP2, and NPHP5, while the second complex consists of NPHP4, NPHP6, and NPHP8.

## Methods

### Animals

Animal experiments were performed in accordance with the guidelines of the Institute for Laboratory Animal Research (Guide for the Care and Use of Laboratory Animals). Prior approval for animal studies was obtained from the University of Michigan Animal Care and Use Committee. ARVO's guidelines were followed for the care and use of animals. The animals were fed ad libitum and were kept on a 12 h light and 12 h dark cycle. The background of the mice is C57BL6/J.

### Antibodies and reagents

RPGR, NPHP5, and CEP290/NPHP6 antibodies have been previously described [19,30,32]. Anti-NPHP1 antibody was purchased from Abcam. Mouse anti-NPHP2/Inversin was procured from Novus Biologicals (Littleton, CO) and mouse anti-NPHP4 was obtained from Abnova (Taipei City, Taiwan).

### Immunoprecipitation

Co-IP experiments were performed as described [19]. Briefly, bovine (Detroit, MI) or mouse retinal extracts were prepared in phosphate buffered saline (KH_2_PO_4_: 1.76 mM; Na_2_HPO_4_: 10 mM; KCl: 2.7 mM; NaCl: 138 mM; pH: 7.4) followed by incubation with the primary antibody or normal IgG (pre-immune bleed of rabbits) overnight at 4 °C. The protein-antibody complexes were then incubated with protein A or protein G agarose beads for 30 min at room temperature with gentle shaking. The beads were then washed with phosphate buffered saline with 1% (V/V) Triton X-100 precipitated proteins and subjected to tandem mass spectrometry. Dissection of complexes by immunodepletion (ID) was performed as described [36,37]. In brief, retina protein extract was subjected to immunoprecipitation using first primary antibody to deplete the associated complexes. The supernatant of the immunoprecipitation was then incubated with the second primary antibody and the precipitated proteins were analyzed by immunoblotting.

### In vitro transcription/translation and glutathione S-transferase pull down

Proteins were synthesized in vitro using the Promega (Madison, WI) TnT Quick kit in the presence or absence of ^35^S-labeled methionine. Glutathione S-transferase (GST) or GST-RLD proteins were purified from *E. coli* and used in the GST pull-down assay, as described [19]. The ^35^S-labeled protein signal was analyzed by autoradiography with STORM 840 (GE Healthcare, Piscataway, NJ). RPGR-RLD was cloned into pGEX4T-2 (GE Healthcare, Piscataway, NJ). cDNA encoding NPHP1, NPHP4 and deleted domains were cloned into plasmid pcDNA3.1 (Invitrogen, Carlsbad, CA) [19].

## Results

### Retinitis pigmentosa GTPase regulator associates with nephrocystin-4

**Figure 1:**
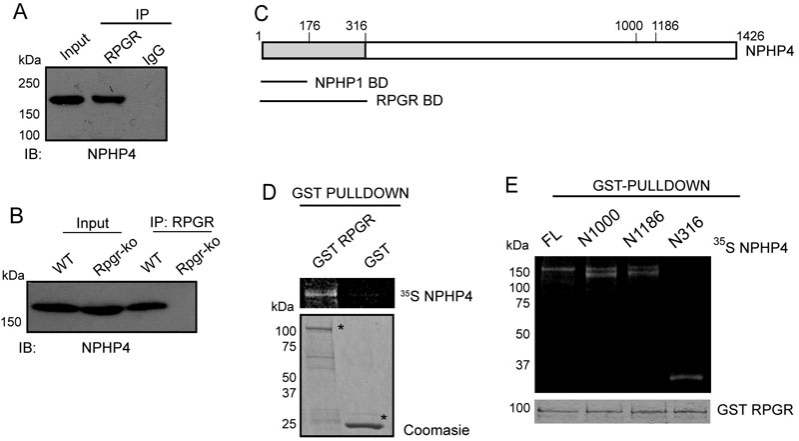
RPGR interacts with NPHP4. **A**: Bovine retinal lysate was subjected to IP using RPGR (**A**) antibody or control IgG (IgG from pre-immune bleed of rabbits) followed by immunoblotting using anti-NPHP4 antibody. The input lane represents 20% of the protein used for IP. **B**: The NPHP4-RPGR complex is disrupted in *Rpgr*-ko retinas: Protein lysates from *wt* or *Rpgr*-ko retinas were immunoprecipitated with RPGR antibody and analyzed by immunoblotting with NPHP4 antibody. Lanes are indicated. **C**: Schematic representation of the primary structure of NPHP4. BD represents the binding domain. **D**: Interaction of GST-RPGR with ^35^S in-vitro translated NPHP4 was analyzed by GST pull-down assay, as described in the experimental procedures. Purified GST moiety was used as control. The lower panel shows Coomassie blue stained gel of the GST-RPGR and GST protein (asterisks) used in the assay. **E**: The GST pull-down assay was performed using GST-RPGR and ^35^S-labeled deletion mutants of NPHP4. The lower panel shows Coomassie blue staining of the GST-RPGR protein used in this assay. Molecular markers in kDa are shown on the left.

To identify RPGR-interacting proteins in mammalian retinas, we performed IP using anti-RPGR antibody followed by mass spectrometry analysis of the precipitated proteins. Our analysis revealed nephroretinin (NPHP4) as a strong interactor of RPGR. NPHP4 is a cilia-centrosomal protein mutated in nephronophthisis and SLSN [38,39]. Given that NPHP4 interacts with RPGRIP1 and NPHP8, and that it localizes to photoreceptor cilia [26,40], we focused on further analyzing the physiologic relevance of RPGR-NPHP4 interaction. Co-IP using anti-RPGR antibody followed by detection of the NPHP4 immunoreactive band further confirmed that RPGR and NPHP4 exist in the same complex in the retina (Figure 1A). Reverse IP using the anti-NPHP4 antibody also revealed RPGR-immunoreactive bands (data not shown). IP using IgG did not show any immunoreactivity. To examine whether RPGR-NPHP4 interaction is relevant to disease, we assessed the association of RPGR and NPHP4 in the *Rpgr*-ko mouse retina. As we have shown before, a deleted variant of RPGR is still expressed in the *Rpgr*-ko mouse retina and can be detected using the RPGR^ORF15^ antibody [19]. Using co-IP, we showed that the mutant RPGR protein cannot associate with NPHP4 in the retina. (Figure 1B). These results suggest that the RLD of RPGR is involved in its interaction with NPHP4.

### Retinitis pigmentosa GTPase regulator directly interacts with nephrocystin-4

To test whether RPGR-RLD interacts directly with NPHP4, we performed a GST pull-down assay using recombinant GST-RLD of RPGR and in vitro translated NPHP4 (full length and different domains [Figure 1C]). Using GST-RLD protein purified from *E. coli* and ^35^S-NPHP4, we found that the RLD directly interacts with NPHP4 (Figure 1D). No interaction was detected when GST alone was used in this experiment. We then sought to identify the domain of NPHP4 that interacts with RPGR-RLD. To this end, we synthesized different ^35^S-labeled domains of NPHP4 (Figure 1C) and used in the GST pull-down assay. As shown in Figure 1E, GST-RLD can interact directly with N-terminal 316 amino acids (N316). An interaction is also detected with the N1000- and N1186 domains of NPHP4.

### Retinitis pigmentosa GTPase interacts with nephrocystin-1

**Figure 2:**
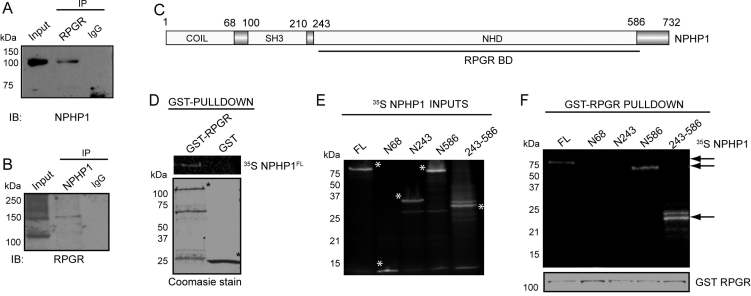
RPGR interacts with NPHP1. **A**, **B**: The association of RPGR with NPHP1 in bovine retina was analyzed by co-IP using appropriate antibodies. Precipitated proteins were analyzed by SDS–PAGE followed by immunoblotting with the indicated antibodies. **C**: Schematic representation of the primary structure of NPHP1. Abbreviations: NHD represents the nephrocystin homology domain; BD represents the binding domain; SH3 represents the Src homology domain. **D**: Interaction of GST-RPGR with ^35^S NPHP1 was analyzed by GST pull-down assay. Amount of GST bound proteins was evaluated by Coomassie blue staining (asterisk; lower panel). **E**: This panel denotes expression of different domains of ^35^S-NPHP1 (asterisks) used in the GST pull-down assay. Gels were analyzed by autoradiography. **F**: The interaction of different domains of NPHP1 with RPGR was analyzed by GST pull-down assay. Coomassie blue staining of the gels loaded with GST-RPGR protein was performed to evaluate the amount of protein in each experiment (lower panel). Molecular weight markers in kDa are shown on the left. Arrows indicate the specific protein bands representing the ^35^S-NPHP1 deleted domains.

It has been shown that NPHP4 interacts with NPHP1 (nephrocystin) [41], which is a ciliary protein mutated in nephronophthisis type 1 [42,43] and which localizes to photoreceptor CC [44]. As NPHP4’s RPGR-binding domain coincides with its NPHP1-binding domain [41], we tested whether RPGR also interacts with NPHP1. Using bovine retinal extract, we found that the anti-RPGR antibody could immunoprecipitate NPHP1 (Figure 2A). Reverse IP using anti-NPHP1 antibody also revealed RPGR-immunoreactive bands (Figure 2B). We then examined the interaction of RPGR with NPHP1 in vitro. Using ^35^S-labeled NPHP1 (full-length and different domains [Figure 2C]) and GST-RLD fusion protein, we showed that full-length NPHP1 directly binds to RLD. No interaction is detected with GST alone (Figure 2D). We also synthesized ^35^S-labeled NPHP1 domains (Figure 2E) and used them in the GST-pull down assay. We found that amino acids 243–586 of NPHP1 were sufficient bind to RPGR-RLD (Figure 2F).

### Retinitis pigmentosa GTPase regulator forms distinct complexes with nephrocystin proteins

**Figure 3:**
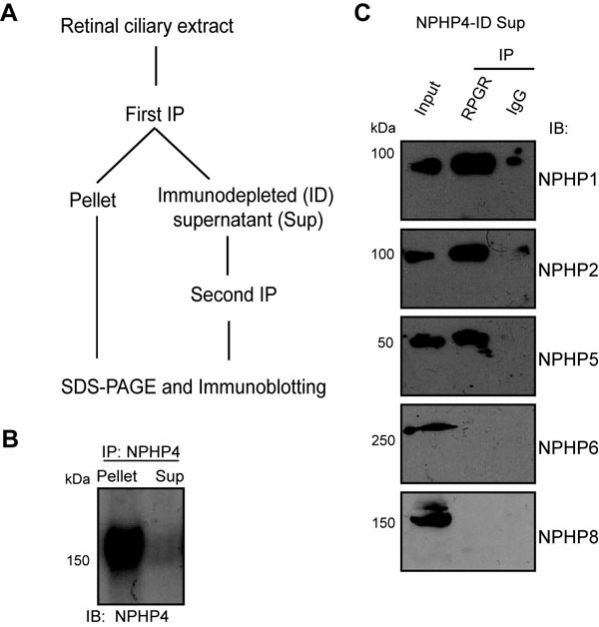
Immunodepletion of NPHP4 in bovine retina. **A**: Schematic representation of the procedure used for ID/IP experiments. **B**: About 500 mg of bovine retinal lysate was subjected to IP using anti-NPHP4 antibody. Precipitated (pellet) as well as supernatant samples were analyzed by immunoblotting using NPHP4 antibody. **C**: NPHP4-immunodepleted supernatant (NPHP4-ID sup) was subjected to IP with anti-RPGR antibody followed by immunoblot analysis using the indicated antibodies. Molecular weight markers in kDa are shown on the left.

**Figure 4:**
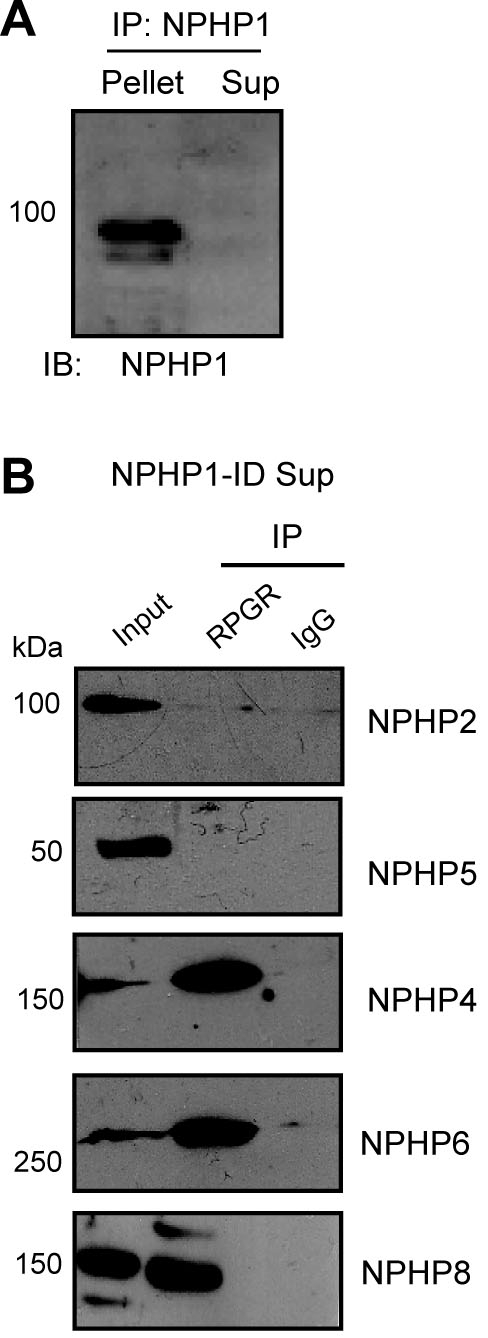
Immunodepletion of NPHP1 in bovine retinas. **A**: NPHP1-containing complexes were immunodepleted from bovine retinal lysates, as described above. The supernatant (sup) was subsequently used for IP experiments. **B**: NPHP1-immunodepleted supernatant (NPHP1-ID sup) was subjected to IP with anti-RPGR antibody followed by immunoblot analysis. Lanes are indicated. Molecular weight markers in kDa are shown on the left.

All NPHP proteins identified to date localize to cilia and centrosomes. In fact, NPHP4 and NPHP1, identified in this study as binding partners of RPGR, have also been localized to photoreceptor CC [40,44,45]. As RPGR is a cilia-centrosomal protein and associates with the majority of NPHP proteins in the retina (NPHP1, NPHP2, NPHP4, NPHP5, NPHP6, NPHP8, and NPHP11) [46], we sought to determine how RPGR might be partitioned in the NPHP complexes. We first performed serial ID of NPHP4 or NPHP1 to immunodeplete the fraction of RPGR that is in complex with these proteins. The remaining RPGR in the supernatant was immunoprecipitated using anti-RPGR antibody and both precipitates were tested for the presence or absence of RPGR or other NPHP proteins. This strategy has been described previously [36,37] and is schematically represented in Figure 3A. We first tested that the anti-NPHP4 antibody is efficient in immunodepleting NPHP4. Using anti-NPHP4 antibody, we found that a majority (>90%) of NPHP4 is immunodepleted from the bovine retinal extracts (Figure 3B; sup represents supernatant). Using the NPHP4-immunodepleted supernatant (NPHP4-ID sup), we performed IP using anti-RPGR antibody and analyzed the precipitate for the presence of other NPHP proteins. We found that the remaining RPGR still associated with NPHP1, NPHP2, and NPHP5. However, NPHP6 and NPHP8 were no longer detected in complex with RPGR in the sample depleted of NPHP4 complexes (Figure 3C). Similar results were obtained when NPHP2 and NPHP5 were immunodepleted from bovine retinal extracts (data not shown). On the other hand, after ID of NPHP1 (Figure 4A), RPGR still associated with NPHP4, NPHP6, and NPHP8, but not with NPHP2 or NPHP5 (Figure 4B). Taken together, our data indicate that there are at least two different RPGR-NPHP complexes: (i) RPGR-NPHP1-NPHP2-NPHP5 and (ii) RPGR-NPHP4-NPHP6-NPHP8.

### Retinitis pigmentosa GTPase regulator-nephrocystin-4 complex is distinct from nephrocystin-1-nephrocystin-4 complex

**Figure 5:**
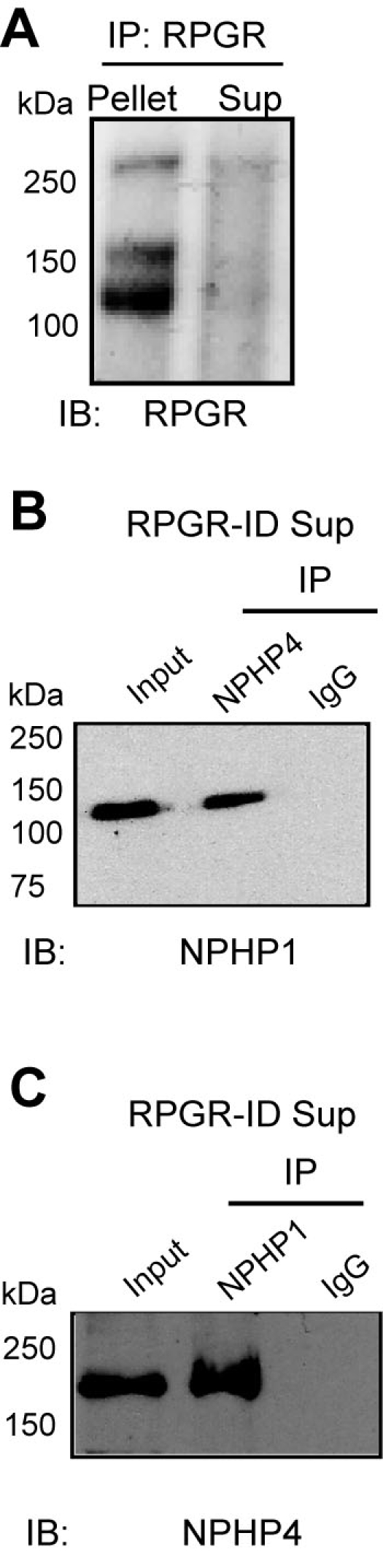
NPHP1 and NPHP4 interact independently of RPGR. **A**: Bovine retinal lysates were subjected to IP using anti-RPGR antibody followed by immunoblotting of the precipitated proteins and supernatant (sup). **B**, **C**: RPGR-immunodepleted RPGR supernatant (RPGR-ID sup) was immunoprecipitated with either anti-NPHP4 (**B**) or anti-NPHP1 (**C**) antibodies and analyzed by SDS–PAGE and immunoblotting. Lanes are indicated. Molecular weight markers in kDa are shown on the left.

We have found that the previously reported NPHP1-binding domain of NPHP4 coincides with its RPGR-binding domain. Hence, we hypothesized that RPGR-NPHP4 complexes are distinct from NPHP4-NPHP1 complexes. As shown above, after serial ID of NPHP1, NPHP4 can still associate with RPGR. We then examined whether ID of RPGR abolishes the NPHP1-NPHP4 complexes. Our analysis revealed that even when the majority of RPGR was depleted, NPHP4 still associated with NPHP1 (Figure 5A-C). These results indicate that there are potentially RPGR-independent NPHP1-NPHP4 complexes in the retina.

## Discussion

Ciliopathies are accompanied by overlapping as well as somewhat distinct phenotypes, although they arise from a common defect in ciliary functions [47]. Nephronophthisis, a ciliopathy characterized by fibrocystic renal disease, is the leading genetic cause of end stage renal disease in children and young adults [6]. The syndromic disorder SLSN is characterized by nephronophthisis and retinal degeneration. To date, 10 NPHP genes have been identified as causing NPHP or SLSN of variable severities (NPHP1–9 and NPHP11) [26,27,29,32,38,42,43,48–52]. All NPHP proteins localize to primary cilia and photoreceptor sensory cilia [5,6,53]. Degeneration of photoreceptors is a commonly observed phenotype in ciliopathies. However, the mechanism of the progression and pathogenesis of photoreceptor degeneration due to mutations in ciliary proteins is not completely understood. Here, we describe the network of ciliary complexes of RPGR and NPHP proteins in mammalian retina that may be altered in disease condition. Our work has revealed important findings. First, we described NPHP4 and NPHP1 as novel RPGR-interacting proteins in the retina. Second, we showed that RPGR and NPHP proteins form distinct complexes in the retina. Third, we demonstrated that the RLD of RPGR might mediate RPGR-NPHP interactions. Significantly, our results also demonstrated that RPGR-NPHP interactions are altered in the mouse mutant of *Rpgr*. As RPGR and its interacting proteins have been shown to play a key role in regulating protein trafficking in photoreceptors, our data point to a role of RPGR-NPHP protein complexes in cilia-dependent transport and associated disease.

It has been shown that NPHP1 and NPHP4 interact with each other in renal epithelial cells [41]. Although RPGR interacts with both NPHP1 and NPHP4, our data suggest that RPGR-NPHP4 and RPGR-NPHP1 complexes may be distinct from the NPHP1-NPHP4 complexes in the retina. We have recently shown that knockdown of RPGR results in shorter cilia in the Kupffer’s vesicles of zebrafish [54]. Interestingly, Mollet et al. [41] reported that knockdown of NPHP1 or NPHP4 also results in shorter cilia. Cumulatively, these studies provide further evidence for a functional overlap between RPGR and NPHP proteins in regulating photoreceptor function.

What is the function of RPGR-NPHP complexes? As photoreceptors rely almost completely on the primary cilium for microtubule-based intersegmental transport of proteins involved in phototransduction and outer segment renewal [22,55–58], slight perturbations in protein trafficking can lead to photoreceptor degeneration and blindness [30,59–61]. Our data suggest that distinct protein complexes may facilitate the binding, trafficking, and release of the cargo moieties at different steps of the protein transport pathways. A communication or “hand over” mechanism may exist between the distinct protein complexes for efficient cargo transfer and delivery. RPGR seems to play a key role in regulating a subset of these complexes in photoreceptors. The degree of disruption of individual complexes at particular steps of the protein transport pathways may determine the severity of associated disease. Further studies are required to assess the physiologic relevance and functionality of these complexes in protein trafficking in photoreceptors.

Ciliopathies exhibit clinical heterogeneity even within single families. The present studies suggest the involvement of genetic modifiers of the disease phenotype. We recently showed that mutations in NPHP8, which alter its interaction with RPGR, act as modifiers of the expressivity of the retinal degeneration phenotype in ciliopathy patients [31]. More recently, it has been reported that mutations in Abelson helper integration site 1 protein homolog (AHI1), which interacts with NPHP1 [62], act as modifier of retinal degeneration phenotype in nephronophthisis [63]. Taken together, the RPGR-interactome dissected in this study provides clues to further analyze the molecular mechanisms underlying the genetic and clinical heterogeneity associated with ciliopathies.
